# Palliative chemotherapy beyond three courses conveys no survival or consistent quality-of-life benefits in advanced non-small-cell lung cancer

**DOI:** 10.1038/sj.bjc.6603383

**Published:** 2006-10-03

**Authors:** C von Plessen, B Bergman, O Andresen, R M Bremnes, S Sundstrom, M Gilleryd, R Stephens, J Vilsvik, U Aasebo, S Sorenson

**Affiliations:** 1 Department of Thoracic Medicine, Haukeland University Hospital and Institute of Medicine, University of Bergen, N-5018 Bergen, Norway; 2 Department of Respiratory Medicine, Sahlgrenska University Hospital and Göteborg University, Gothenburg, Sweden; 3 Department of Oncology, University of Tromsø and University Hospital of Northern Norway, Tromsø, Norway; 4 Department of Oncology, St Olavs University Hospital, Trondheim, Norway; 5 Department of Medical Oncology, Cancer Division, MRC Clinical Trials Unit, St Bartholomew's Hospital, London, UK; 6 Department of Lung and Occupational Medicine, St Olavs University Hospital, Trondheim, Norway; 7 Department of Medicine, University of Tromsø and University Hospital of Northern Norway, Tromsø, Norway

**Keywords:** chemotherapy, non-small-cell lung cancer, palliative, treatment duration, quality of life, survival

## Abstract

This randomised multicentre trial was conducted to establish the optimal duration of palliative chemotherapy in advanced non-small-cell lung cancer (NSCLC). We compared a policy of three *vs* six courses of new-generation platinum-based combination chemotherapy with regard to effects on quality of life (QoL) and survival. Patients with stage IIIB or IV NSCLC and WHO performance status (PS) 0–2 were randomised to receive three (C3) or six (C6) courses of carboplatin (area under the curve (AUC) 4, Chatelut's formula, equivalent to Calvert's AUC 5) on day 1 and vinorelbine 25 mg m^−2^ on days 1 and 8 of a 3-week cycle. Key end points were QoL at 18 weeks, measured with EORTC Quality of Life Questionnaire (QLQ)-C30 and QLQ-LC13, and overall survival. Secondary end points were progression-free survival and need of palliative radiotherapy. Two hundred and ninety-seven patients were randomised (C3 150, C6 147). Their median age was 65 years, 30% had PS 2 and 76% stage IV disease. Seventy-eight and 54% of C3 and C6 patients, respectively, completed all scheduled chemotherapy courses. Compliance with QoL questionnaires was 88%. There were no significant group differences in global QoL, pain or fatigue up to 26 weeks. The dyspnoea palliation rate was lower in the C3 arm at 18 and 26 weeks (*P*<0.05), but this finding was inconsistent across different methods of analysis. Median survival in the C3 group was 28 *vs* 32 weeks in the C6 group (*P*=0.75, HR 1.04, 95% CI 0.82–1.31). One- and 2-year survival rates were 25 and 9% *vs* 25 and 5% in the C3 and C6 arm, respectively. Median progression-free survival was 16 and 21 weeks in the C3 and C6 groups, respectively (*P*=0.21, HR 0.86, 95% CI 0.68–1.08). In conclusion, palliative chemotherapy with carboplatin and vinorelbine beyond three courses conveys no survival or consistent QoL benefits in advanced NSCLC.

During the last decade, chemotherapy has become widely used in the palliative treatment of patients with advanced non-small-cell lung cancer (NSCLC). Today, it is acknowledged that platinum-based chemotherapy improves quality of life (QoL) (Helsing *et al*, 1998; Cullen *et al*, 1999; Gridelli *et al*, 1999; Thongprasert *et al*, 1999; Anderson *et al*, 2000; Ranson *et al*, 2000) and increases survival in patients with a good performance status (PS) by 2–3 months (Marino *et al*, 1994; Anonymus, 1995; Souquet *et al*, 1995; Spiro *et al*, 2004). Two-drug platinum-based regimens have proven superior to single drug therapy and equivalent in efficacy to three-drug regimens (Alberola *et al*, 2003; Delbaldo *et al*, 2004). Given potential side effects, time spent in hospital, the cost of patient care, interindividual response variation and the palliative nature of the chemotherapy, it is clinically highly relevant to establish the optimal treatment duration. Evidence concerning this question is scarce. Two trials of induction *vs* induction plus maintenance treatment in responding patients with advanced NSCLC suggested that treatment could be confined to two or three cycles (Buccheri *et al*, 1989; Weynants *et al*, 1997), but none of these trials specifically addressed the question of treatment duration. An expert panel at the American Society of Clinical Oncology in 1997 (Anonymus, 1997) recommended a maximum of eight chemotherapy courses in patients with stage IV NSCLC. The 2003 update of the guidelines recommended limiting chemotherapy to six cycles in general and stopping treatment after four cycles in stage IV patients who do not respond to treatment. These recommendations were based on a trial comparing three *vs* six courses of mitomycin, vinblastine and cisplatin (Smith *et al*, 2001), and a study that compared four courses of carboplatin/paclitaxel with the same combination given until progression (Socinski *et al*, 2002). Neither trial showed benefits from longer treatment duration.

The current trial was designed to compare a treatment policy of three (C3) *vs* six (C6) cycles of a modern platinum-based two-drug combination regimen. The main research question was whether six courses would be superior to three courses with respect to QoL and survival outcomes.

## MATERIALS AND METHODS

### Patients

Patients with cytologically or histologically verified NSCLC stage IIIB or IV who were not candidates for treatment with a curative intent and who had a WHO PS of 0, 1 or 2 were eligible. No upper age limit was defined. A white blood cell (WBC) count >3.0 × 10^9^ l^−1^, platelet count >100 × 10^9^ l^−1^, serum creatinine <1.5 times the upper reference limit, and bilirubin, ASAT and ALAT less than twice the upper reference limit were required. Exclusion criteria were other active malignancy, pregnancy or breast feeding. Patients had to be chemotherapy naïve and had to understand oral and written information.

### Baseline investigations

Demographic and clinical data (age, gender, histological or cytological tumour type, PS, disease stage, body height and weight) as well as laboratory measures (haemoglobin, leucocyte and platelet counts, sodium, potassium, calcium, albumin, ASAT, ALAT, bilirubin, alkaline phosphatase, lactate dehydrogenase and creatinine) were recorded. All patients had to complete the first QoL questionnaire before randomisation.

### Chemotherapy

Patients were randomised to receive three (C3) or six (C6) courses of intravenous (i.v.) carboplatin and vinorelbine. Both drugs were administered on day 1, generally on an outpatient basis, and vinorelbine was repeated on day 8 of each 3-week cycle. Haematologic parameters were measured before each chemotherapy administration and on day 15. The dose of carboplatin was calculated by means of Chatelut's formula (area under the curve (AUC)=4), and the vinorelbine dose was 25 mg m^−2^. Vinorelbine, diluted in 100 ml glucose 50 mg ml^−1^, was given as a 10 min infusion, and carboplatin, diluted in 500 ml glucose 50 mg ml^−1^, was infused during 1 h. Area under the curve=4 in Chatelut's formula corresponds to AUC=5 in Calvert's. Immediately before chemotherapy on day 1, patients received antiemetic prophylaxis with dexamethasone or betametasone 8 mg i.v. and ondansentron 8 mg (alternatively tropisetron 5 mg) i.v. Antiemetic prophylaxis on day 8 was optional.

In case of moderate haematological toxicity (WBC 2.5–2.9 × 10^9^ l^−1^ and/or platelets 75–99 × 10^9^ l^−1^) on day 1 of subsequent treatment courses, the doses of carboplatin and vinorelbin were reduced by 33%. In case of more pronounced haematological toxicity (WBC <2.5 × 10^9^ l^−1^ and/or platelets <75 × 10^9^ l^−1^), the next chemotherapy course was postponed for 1 week and all subsequent courses remained reduced by 33%. In patients with leukopenia-associated infection chemotherapy was postponed until clinical recovery and normalised WBC and platelets. At continuation, further doses were reduced by 33%. Treatment was prematurely discontinued in case of disease progression, unacceptable toxicity, or on the patient's request.

### Randomisation

First patients signed the informed consent form and completed the baseline QoL questionnaire. Then they were randomised after stratification by PS (0–1 *vs* 2) and by institution using a minimisation process with a probability of 0.75 (Pocock, 1985).

### Assessment of QoL

Quality of life was measured by the patient-completed EORTC Core Quality of Life Questionnaire (QLQ-C30 version 2) (Aaronson *et al*, 1993) and the lung cancer-specific module QLQ-LC13 (Bergman *et al*, 1994). The QLQ-C30 measures functional aspects of QoL and symptoms commonly reported in cancer patients, while the QLQ-LC13 addresses symptoms specifically associated with lung cancer and its treatment. In the present trial, dyspnoea, pain, fatigue and global QoL were predefined as the primary QoL outcome measures. Quality of life assessments were scheduled at baseline and then at weeks 3, 6, 9 (end of C3 study treatment), 12, 15, 18 (end of C6 study treatment), 26, 34, 42 and 50 in both study arms. In this study, QoL questionnaires up to 26 weeks were used for analysis. Follow-up questionnaires were either administered at the scheduled outpatient visits (Sweden) or mailed directly from the study office to the patients (Norway). Patients received one mailed reminder after 14 days if the questionnaire was not returned.

### Assessment of toxicity

At the first visit in each treatment cycle and at the 8-weekly follow-up visits, patients underwent clinical examination with an evaluation of PS, weight and blood tests. Local investigators recorded nadir values of haemoglobin, leucocyte and platelet counts, the number of transfusions, leukopenic infections, thrombocytopenic bleedings, events of phlebitis during the study treatment period and hospital admissions due to chemotherapy side effects. After a patient's death or at registration cutoff in June 2004, investigators also reported additional treatments such as radiation, second-line chemotherapy or surgery as well as the cause of death. Mortality data were verified by the national mortality registries.

### Assessment of disease progression

Tumour status was evaluated by using chest X-ray, performed in both treatment arms at baseline, after completion of the scheduled courses, and then every eighth week or at the investigators' discretion. Disease progression was defined either as an increase of the longest measurable tumour diameter by at least 20% compared to the minimum length after treatment start, or by the occurrence of new metastases, or death with residual tumour.

### Study end points

Main end points of the study were QoL at 18 weeks and overall survival. Further end points were progression-free survival and need of palliative radiotherapy.

### Statistical considerations

Sample size was estimated to detect a mean global QoL difference at 18 weeks of 11 score points or more on the QLQ-LC30 scale ranging from 0 to 100, which is considered a clinically significant score difference (King, 1996; Osoba *et al*, 1998). With an s.d. of 23 (Aaronson *et al*, 1993), a type I error of 5% and power of 80% using a two-sided *t*-test, these criteria required 70 evaluable patients in each group. Assuming a drop-out rate by 18 weeks of about 50% due to disease progression or death, 300 randomised patients were considered necessary.

With respect to chemotherapy-related haematological side effects, the maximum toxicity ever experienced during treatment is reported. Differences between treatment arms were tested with a *χ*^2^ test. Survival time was measured from the date of randomisation. Survival curves were calculated by the method of Kaplan and Meier and compared by the log-rank test. Additionally, a Cox model was used for estimation of hazard ratios. All data analyses followed the intention-to-treat principle.

Quality of life outcomes were analysed in four ways: (1) group comparison of scale scores at each time point, (2) score changes from baseline, (3) AUC and (4) rates of symptom palliation defined as improvement, control or prevention, and death counted as nonpalliation (Stephens *et al*, 1999). Improvement was defined as a change in reported baseline symptom levels from moderate or severe (67–100 points) to none or little (0–33 points) (Langendijk *et al*, 2000), or from little to no symptoms without subsequent deterioration by the time of group comparison. Control of symptoms was defined as stable symptom levels between 1 and 33 points, while prevention of symptoms was assumed when patients did not report symptoms during the study period. A similar approach was used for the global QoL scale. Here, four score level ranges were defined: 0–24, 25–49, 50–74 and 75–100, with 0 being the worst and 100 the best possible score.

Main time point for the analysis of the QoL outcome was at 18 weeks, and assessment at 26 weeks was considered important for confirmatory analyses. Assessment at 9 weeks served as quality control of treatment arm balance. Nonparametric tests were used for group comparisons. Group differences that were consistent across methods of analysis or detected with a *P*-value of 0.01 or less were interpreted as probable treatment effects.

### Ethical considerations

The study protocol was approved by the Regional Ethical Committee of Western Norway and at the University of Gothenburg, Sweden.

## RESULTS

### Patient characteristics

From May 2000 until March 2002, 300 patients were randomised at five university hospitals and 20 regional and local hospitals in Norway (24 centres, *N*=262) and Sweden (one centre, *N*=38). Three patients with thyroid, small-cell lung and peritoneal cancer, respectively, were misdiagnosed and excluded from the study, leaving 297 patients (C3 150, C6 147). Median follow-up time by June 2004 was 36 months. Patients' pretreatment characteristics are presented in Table 1. The treatment arms were well balanced with regard to age, PS, stage and histology, while the C6 arm comprised a larger proportion of females.

### Compliance with chemotherapy

Completion of all planned chemotherapy courses was reported in 117 (78%) and 79 (54%) patients in the C3 and C6 arm, respectively (Table 2). The mean and corresponding median numbers of courses were 2.7 and 3, respectively, in the C3 arm and 4.5 and 6, respectively, in the C6 arm. Vinorelbine on day 8 was omitted at least once in five patients in the C3 arm and in 20 patients in the C6 group.

### Compliance with QoL questionnaires

Two hundred and ninety-seven patients returned 1715 completed questionnaires (of 1911 expected, deceased patients excluded) during the initial 26 weeks study period. Thirty questionnaires were not adequately filled in, leaving 1685 evaluable questionnaires (C3, *N*=826; C6, *N*=859). Thus, the compliance rate with QoL questionnaires during the study period was 88% (C3 86%; C6 91%).

### QoL outcome

Scores for global QoL as well as dyspnoea, pain and fatigue at baseline, 9, 18 and 26 weeks are displayed in Table 3.

C6 patients reported lower dyspnoea scores, as measured by the three-item QLQ-LC13 dyspnoea scale, at 18 and 26 weeks than did the C3 patients. These group differences were not seen with the single-item QLQ-C30 dyspnoea measure. No significant group differences were seen at any time point for pain, fatigue or global QoL.

**Figure 1:**
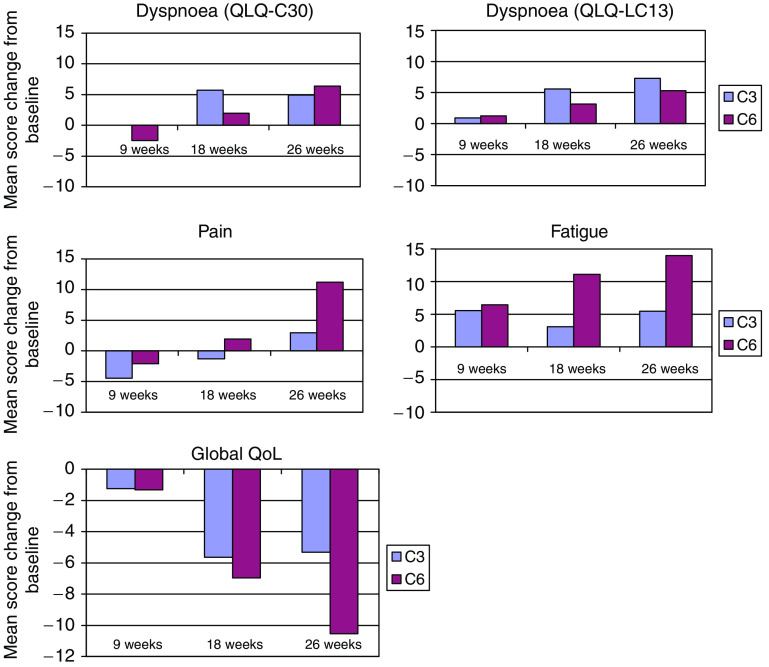
Mean score changes from baseline to key follow-up time points for primary outcome symptoms and global QoL, calculated from individual patients measured at baseline and 9 weeks (C3, *N*=105; C6, *N*=103), 18 weeks (C3, *N*=77; C6, *N*=87) and 26 weeks (C3, *N*=62; C6, *N*=73), respectively. For symptom measures score, changes >0 indicate increased symptoms (i.e. deterioration), while for the global QoL score, changes >0 indicate improvement. No significant group differences were seen.

Mean score changes from baseline to 9, 18 and 26 weeks are shown in Figure 1.

The changes of dyspnoea scores suggested a small symptom increase over time in both treatment groups, with no significant group differences at 18 or 26 weeks. Changes of pain scores did not differ up to 18 weeks, whereas a trend towards a less favourable outcome in the C6 group at 26 weeks was detected (*P*=0.08). Similarly, fatigue scores tended to increase somewhat more in the C6 group at 18 (*P*=0.13) and 26 weeks (*P*=0.09). Global QoL tended to deteriorate over time in both C3 and C6 patients. However, score changes did not differ significantly between the groups.

Analysis of AUC up to 18 and 26 weeks did not reveal any significant group differences in any of the core symptom items or global QoL (data not shown).

**Figure 2:**
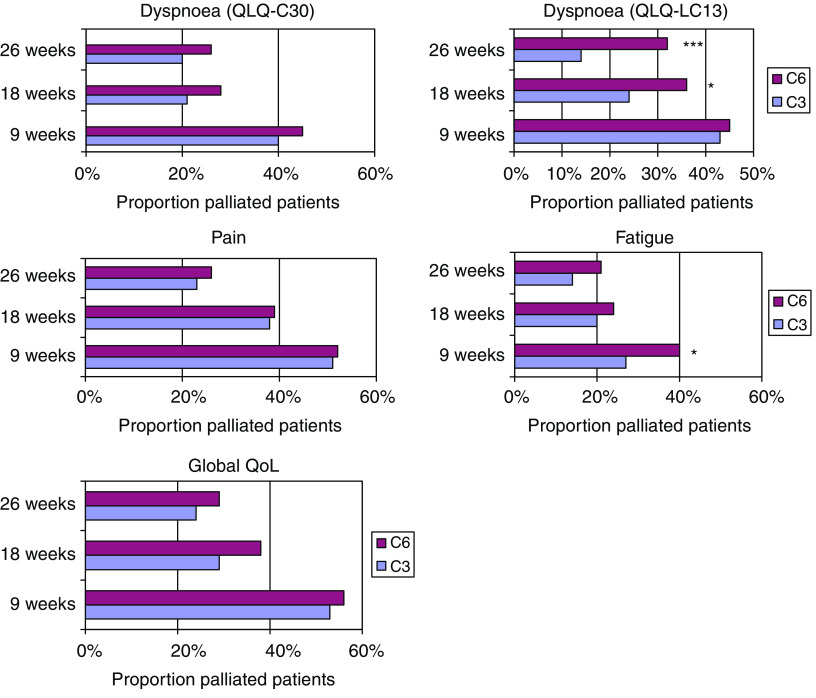
Rates of palliation of core symptoms and global QoL, defined as improvement, control or prevention, and death counted as nonpalliation. Numbers of evaluable patients (including deceased) were 256 at 9 weeks (C3 127, C6 129), 270 at 18 weeks (C3 133, C6 137) and 282 at 26 weeks (C3 140, C6 142). ^*^P<0.05; ^***^P<0.001; *χ*^2^ test.

Finally, palliation rates of the core symptoms and global QoL are displayed in Figure 2.

Palliation rates varied from 34% (fatigue) to 55% (global QoL) by 9 weeks (C3 and C6 combined), but declined over time. A significantly larger proportion of C6 than C3 patients were still palliated with regard to dyspnoea at 18 (*P*<0.05) and 26 (*P*<0.001) weeks when measured by the three-item QLQ-LC13 dyspnoea scale, but this group difference could not be detected with the single-item QLQ-C30 dyspnoea measure. As for the fatigue measure, there seemed to be a palliation advantage for the C6 group at 9 weeks (*P*<0.05), which could hardly be attributable to treatment effects. By the 18 and 26 weeks follow-up measurements, there were no significant group differences in palliation of fatigue, pain or global QoL.

With regard to the remaining QoL measures, which were used for exploratory purposes, there was a larger deterioration in role functioning scores from baseline to 26 weeks in the C6 group (mean score change −19.0 *vs* −7.4 in the C3 group, *P*<0.05) and increased nausea and vomiting by 18 weeks (mean score change +4.2 *vs* −0.4 in the C3 group, *P*<0.05). In the C3 group, there was a larger improvement in the arm/shoulder pain score by 18 weeks (mean score change −10 *vs* −0.8 in the C6 group, *P*<0.05). Otherwise, there were no significant group differences in score levels, score changes, AUC or palliation rates up to 26 weeks.

### Survival

**Figure 3:**
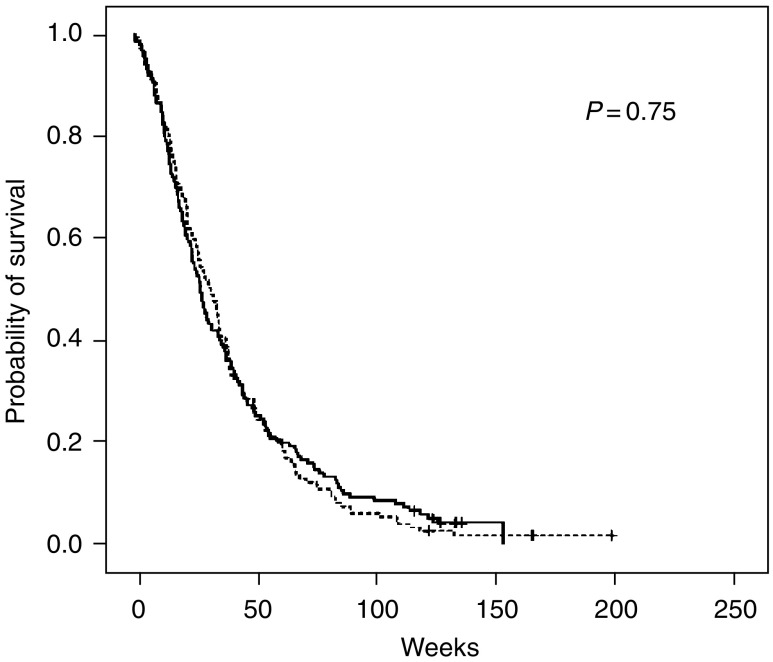
Overall survival by treatment group: C3 (randomised to receive three courses) marked with solid line and C6 (randomised to receive six courses) with dotted line. *P*-value refers to a log-rank test.

Overall survival is shown in Figure 3. Median survival time was 28 and 32 weeks in the C3 and C6 arm, respectively (*P*=0.75). The hazard ratio for the C6 arm was 1.04 (95% CI 0.82–1.31). One- and 2-year survival rates were 25 and 9% *vs* 25 and 5% in the C3 and C6 arm, respectively.

By June 2004, 288 patients were dead. The cause of death was recorded in 275 patients, 254 deaths were related to lung cancer, two to chemotherapy side effects and three were related to side effects of other cancer treatments. Sixteen patients died of other causes than lung cancer or its treatment.

**Figure 4:**
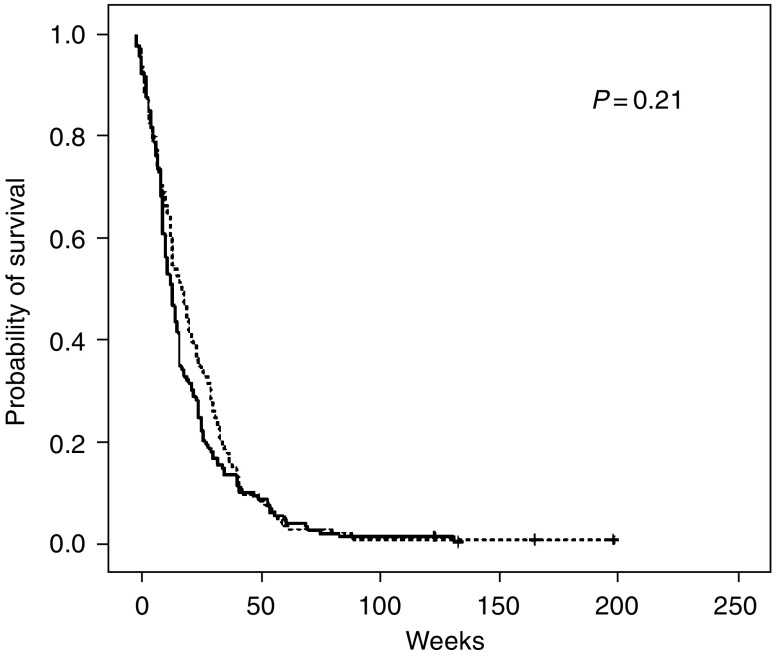
Progression-free survival by treatment group: C3 (randomised to receive three courses) marked with solid line and C6 (randomised to receive six courses) with dotted line. *P*-value refers to a log-rank test.

Progression-free survival time is shown in Figure 4. Median progression-free survival time was 16 and 21 weeks in the C3 and C6 groups, respectively (*P*=0.21). The hazard ratio for the C6 arm was 0.86 (95% CI 0.68–1.08).

### Additional treatment after completed chemotherapy

A total of 285 patients were assessable for additional treatments after completed first-line chemotherapy. Of these patients, 62 (22%) in the C3 arm and 68 (24%) in the C6 arm received palliative irradiation (*P*=0.4). Overall, sixty-four patients received second-line chemotherapy, 35 (12%) in the C3 group and 29 (10%) the C6 group (*P*=0.4).

### Toxicity

Haematological toxicities are presented in Table 4. Approximately one-third of patients in both treatment groups experienced grade 3 or 4 leukopenia.

Other side effects were evaluable in 87% of all patients. In the C3 group, 27 (20%) patients had leukopenic infections compared with 21 (16%) in the C6 group (*P*=0.5). The total number of blood transfusions was 20 (15%) in the C3 *vs* 44 (34%) in the C6 group (*P*=0.003). No thrombocytopenic bleedings were recorded. There was one episode of grade 5 toxicity in each treatment arm. Fourteen (11%) *vs* 18 (14%) in the C3 and C6 group, respectively, had episodes of phlebitis (*P*=0.5). Thirty-two (C3) and 26 (C6) hospital admissions due to chemotherapy side effects were recorded (*P*=0.3).

## DISCUSSION

In this randomised multicentre trial in two Scandinavian countries a treatment policy of six courses of a platinum-based two-drug chemotherapy regimen did not convey any survival or consistent QoL benefits over three courses of the same regimen, with comparable toxicity. These findings challenge recent guidelines that recommend a maximum of six courses of chemotherapy in stage IV NSCLC (Pfister *et al*, 2004).

In this study, group differences in QoL measures were generally small and inconsistent across methods of analysis. The QoL results did not indicate improved pain or fatigue control with the C6 regimen. Neither did patients' functioning or global QoL significantly improve with the longer treatment. On the other hand, the perceived side effects of prolonged treatment were marginal, mainly consisting of moderately increased nausea and vomiting. The only variable that pointed to a potential benefit from prolonged treatment was improved dyspnoea control at 18 and 26 weeks in the C6 *vs* C3 arm when measured by the three-item QLQ-LC13 scale. However, no corresponding difference in dyspnoea control was shown in score changes from baseline or AUC comparison, neither was it reproduced with the single-item QLQ-C30 dyspnoea measure. The latter could possibly be explained by an increased sensitivity to clinical change with the three-item scale (Bergman *et al*, 1994).

The study is representative of the everyday clinical setting with over 20% of all newly diagnosed stage IIIB and IV NSCLC patients in the Norwegian study regions (Cancer Registry of Norway, 2005) participating. Of these patients, 45% were over the age of 65 years compared to 61% in the Norwegian Cancer Registry. Still, the proportion of elderly patients was higher than in typical clinical lung cancer trials (Hutchins *et al*, 1999; Lewis *et al*, 2003; Murthy *et al*, 2004).

At the time this study was planned, single-drug efficacy of vinorelbine had been demonstrated (Gridelli *et al*, 1997) and the combination of carboplatin and vinorelbine was well tolerated in phase II trials (Masotti *et al*, 1995; Pronzato *et al*, 1996; Gridelli *et al*, 1998, 1999; Santomaggio *et al*, 1998). Later vinorelbine/cisplatin regimens have shown similar efficacy when compared to other cisplatin combinations with third-generation cytotoxic drugs (Schiller *et al*, 2002). A recent meta-analysis comparing cisplatin and carboplatin in the treatment of advanced NSCLC found no significant differences in overall survival in spite of a higher response rate for cisplatin. Treatment with cisplatin was associated with more nausea–vomiting than carboplatin (Ardizzoni *et al*, 2006). For the palliative treatment in the unselected population of this study, we assumed equal efficacy of both drugs and chose carboplatin because of its more feasible outpatient administration and more favourable toxicity profile.

The proportion of patients receiving second-line chemotherapy (C3 12%, C6 10%) was small in comparison to a recent survey (Hensing *et al*, 2005) that reported 45% second-line chemotherapy for the patient population of an earlier phase III trial (Socinski *et al*, 2002). However, these figures are representative of the pattern of chemotherapy use for NSCLC in Norway when the study was initiated in 2000. In fact, the study was instrumental in introducing chemotherapy for advanced NSCLC in the country. A consequence of the relatively infrequent use of second-line treatment is that the analysis of overall survival by first-line treatment strategies is less biased.

The evaluation of progression-free survival and chemotherapy toxicity reported by the local investigators was not systematically reviewed and the information on validity of these data is limited. However, the main study end points, overall survival and QoL, were recorded independently of local investigators, and were therefore not affected by this limitation.

During the inclusion phase of the present study, a British group (Smith *et al*, 2001) reported the results from a randomised trial of three *vs* six courses of mitomycin, vinblastine and cisplatin in 308 patients with NSCLC, stage IIIB and IV, and PS 0–2. They found similar median survival times and 1-year survival rates for patients in both study arms, indicating no survival benefit from the longer duration of chemotherapy. Furthermore, QoL and duration of symptom relief were similar in both arms, although patients receiving six courses reported somewhat more pronounced fatigue, nausea and vomiting, probably related to increased treatment toxicity. The two studies were comparable in terms of design and study population, but ours had a higher median age of 65 *vs* 63 years in the British study. Furthermore, treatment compliance was higher in this study, with 78 and 54% completion rate in the C3 and C6 arm, respectively, compared with 72 and 31% in the British trial. This difference probably reflects a better tolerance for the vinorelbine/carboplatin regimen.

In 2002, Socinski *et al* (2002) reported the results from a randomised study comparing four courses of carboplatin/paclitaxel chemotherapy with continuous treatment until progression in 230 patients with NSCLC stage IIIB or IV and PS 0–1. In both arms, the median number of actually delivered chemotherapy courses was 4, with 57% of patients in the short treatment arm receiving all scheduled treatments, and 42% of patients in the continuous treatment arm receiving five or more courses. In spite of their generally more fit study population (no PS 2 patients), prolonged treatment did not yield any benefits in terms of survival, QoL or response rates. An increasing rate of peripheral neuropathy was seen in patients receiving more than four courses.

Limiting the number of chemotherapy courses would reduce total treatment costs (Braud *et al*, 2003) as cytotoxic drugs and their administration constitute 14–20% (Jaakkimainen *et al*, 1990; Billingham *et al*, 2002) of the health-care costs of advanced lung cancer. Furthermore, as many patients spend hours in hospital (Plessen von and Aslaksen, 2005) and travelling back and forth for chemotherapy, a reduction in the number of courses will also directly benefit the patient. Finally, reducing the number of courses can free limited oncological outpatient capacity (Clegg *et al*, 2001).

Around 50% of patients with lung cancer receive chemotherapy during more than 2 months of the final 6 months of their lives, and 10% during their last 4 weeks (Emanuel *et al*, 2003). These proportions are considerable taking into account limited benefits (Spiro and Porter, 2002) and toxicity of chemotherapy. Furthermore, it cannot be expected that patients, after completed treatment, generally have a positive attitude towards chemotherapy (Silvestri *et al*, 1998). On the other hand, it has been shown that patients with cancer are willing to accept toxic therapy for minimal benefits (Slevin *et al*, 1990; Bremnes *et al*, 1995; Hirose *et al*, 2005). It is a challenge for any clinician to give balanced information about potential effects and side effects of palliative chemotherapy and when to stop the treatment (Mitchell and Currow, 2002). The confirmatory evidence from this study, indicating similar QoL and survival after three or six courses of chemotherapy, will hopefully support clinicians and their patients in the decision-making process regarding the duration of palliative chemotherapy, and should be taken into account in clinical guidelines on the palliative treatment of advanced NSCLC.

## Figures and Tables

**Table 1:** Patient characteristics at baseline

	**Treatment group**		
	**C3**	**C6**	**Total**
No. of patients	150	147	297
Sex, M/F	104/46	84/63	188/109
Age, median (range)	64 (35–84)	65 (34–83)	64 (34–84)
			
*PS*			
0	31	26	57
1	76	74	150
2	43	47	90
			
*Stage*			
IIIB	37	34	71
IV	113	113	226
			
*Histology*			
Adenocarcinoma	63	66	129
Squamous cell	46	33	79
Large cell	15	21	36
Adenosquamous	3	0	3
NSCLC – not specified	23	27	50

NSCLC=non-small-cell lung cancer; PS=performance status.

**Table 2:** Numbers and percentages of patients treated at each course

	**Course number**					
	**1**	**2**	**3**	**4**	**5**	**6**
C3 (*N*=150)	150	136	117	—	—	—
Per cent	100	91	78	—	—	—
C6 (*N*=147)	143	134	116	99	88	79
Per cent	97	91	79	67	60	54

C3 patients were randomised to receive three courses, while C6 patients were randomised to receive six courses of chemotherapy

**Table 3:** Mean scores for the primary QoL outcome dimensions at 0, 9, 18 and 26 weeks by treatment group

	**0 weeks**		**9 weeks**		**18 weeks**		**26 weeks**	
	**C3**	**C6**	**C3**	**C6**	**C3**	**C6**	**C3**	**C6**
No. of patients alive	150	147	129	127	96	99	75	80
No. of questionnaires	148	147	105	108	78	87	63	73
Global QoL	50.8	56.7	54.1	58.4	49.6	55.3	49.0	52.4
Dyspnoea (QLQ-C30)	49.0	48.3	46.1	41.7	53.0	47.7	54.5	46.6
Dyspnoea (QLQ-LC13)	37.9	37.8	38.6	35.0	45.1	37.6*	47.9	38.3*
Pain	35.7	30.6	30.6	27.8	29.5	30.7	34.7	38.8
Fatigue	47.2	44.5	49.8	47.1	49.4	50.2	52.1	51.1

QLQ=Quality of Life Questionnaire; QoL=quality of life.

All scale scores range from 0 to 100. For Global QoL, a higher score indicates better QoL, while for the symptom measures, a higher score indicates more pronounced symptoms.

* *P*<0.05; Mann–Whitney *U*-test.

**Table 4:** Numbers (%) of patients in each treatment arm experiencing haematological toxicity by worst CTC grading

	**C3 (*N*=150)**		**C6 (*N*=147)**		
**CTC grade**	**1+2**	**3+4**	**1+2**	**3+4**	***P*-value**
WBC	54 (36)	52 (35)	57 (40)	46 (32)	0.79
Haemoglobin	133 (89)	5 (3)	121 (85)	13 (9)	0.11
Platelets	30 (20)	2 (1)	50 (35)	1 (1)	0.02

CTC=common toxicity criteria; WBC=white blood cell.

*P*-values refer to *χ*^2^ tests.
